# Comparison between cardiac output values measured by thermodilution and partial carbon dioxide rebreathing in patients with acute lung injury

**DOI:** 10.1590/S1516-31802004000600002

**Published:** 2004-11-04

**Authors:** Jorge Luís dos Santos Valiatti, José Luiz Gomes do Amaral

**Keywords:** Cardiac output, Carbon dioxide, Thermodilution, Adult respiratory distress syndrome, Intensive care units, Débito cardíaco, Dióxido de carbono, Termodiluição, Síndrome do desconforto respiratório do adulto, Unidade de terapia intensiva

## Abstract

**CONTEXT:**

Thermodilution, which is considered to be a standard technique for measuring the cardiac output in critically ill patients, is not free from relevant risks. There is a need to find alternative, noninvasive, automatic, simple and accurate methods for monitoring cardiac output at the bedside.

**OBJECTIVE:**

To compare cardiac output measurements by thermodilution and partial carbon dioxide rebreathing in patients with acute lung injury at two levels of severity (lung injury score, LIS: below 2.5, group A; and above 2.5, group B).

**TYPE OF STUDY:**

Comparative, prospective and controlled study.

**SETTING:**

Intensive Care Units of two university hospitals.

**METHODS:**

Cardiac output was measured by thermodilution and partial carbon dioxide rebreathing. Twenty patients with acute lung failure (PaO_2_/FiO_2_ < 300) who were under mechanical ventilation and from whom 294 measurements were taken: 164 measurements in group A (n = 11) and 130 in group B (n = 9), ranging from 14 to 15 determinations per patient.

**RESULTS:**

There was a poor positive correlation between the methods studied for the patients from groups A (r = 0.52, p < 0.001) and B (r = 0.47, p < 0.001). The application of the Bland-Altman test made it possible to expose the lack of agreement between the methods (group A: −0.9 ± 2.71 l/min; 95% CI = −1.14 to −0.48; and group B: −1.75 ± 2.05 l/min; 95% CI = −2.11 to −1.4). The comparison of the results (Student t and Mann-Whitney tests) within each group and between the groups showed significant difference (p = 0.000, p < 0.05).

**DISCUSSION:**

Errors in estimating CaCO_2_ (arterial CO_2_ content) from ETCO_2_ (end-tidal CO_2_) and situations of hyperdynamic circulation associated with dead space and/or increased shunt possibly explain our results.

**CONCLUSION:**

Under the conditions of this study, the results obtained allow us to conclude that, in patients with acute lung injury, the cardiac output determined by partial rebreathing of CO_2_ differs from the measurements obtained by thermodilution. This difference becomes greater, the more critical the lung injury is.

## RESUMO

**CONTEXTO:**

A termodiluição, considerada técnica padrão para medida do débito cardíaco em pacientes graves, não é isenta de riscos relevantes. Faz-se necessário encontrar métodos alternativos não invasivos, automáticos, simples e acurados para monitorar o débito cardíaco à beira do leito.

**OBJETIVO:**

Comparar as medidas do débito cardíaco obtido com os métodos termodiluição e reinalação parcial de gás carbônico em pacientes com lesão pulmonar aguda em dois níveis de gravidade (índice de lesão pulmonar – LIS abaixo de 2,5 grupo A; e acima de 2,5, grupo B).

**TIPO DO ESTUDO:**

Comparativo, prospectivo, controlado.

**LOCAL:**

Unidades de Terapia Intensiva de dois hospitais-escola.

**MÉTODOS:**

Vinte pacientes acometidos de insuficiência respiratória aguda (PaO_2_/FiO_2_ < 300), sob ventilação pulmonar artificial, nos quais foram realizadas 294 medidas, 164 medidas no grupo A (n = 11) e 130 no grupo B (n = 9),variando de 14 a 15 medidas por paciente, foram estudados. Débito cardíaco foi medido com termodiluição e reinalação parcial de gás carbônico.

**RESULTADOS:**

A correlação entre os métodos estudados foi fraca no grupos A (r = 0,52, p < 0,001*) e no B: r = 0,47, p < 0,001*). A aplicação do teste de Bland-Altman permitiu evidenciar a discordância entre os métodos (grupo A: −0,9 ± 2,71 l/min; IC 95% = - 1,14 a −0,48; e grupo B: −1,75 ± 2,05 l/min (IC 95% = −2,11 a −1,4). A comparação dos resultados (testes t para grupos emparelhados e Mann-Whitney) obtidos nos grupos e entre os grupos de estudo revelou diferenças (p = 0,00*, p < 0,05).

**DISCUSSÃO:**

Erros em estimar o CaCO_2_ (conteúdo arterial de CO_2_) através da ETCO_2_ (CO_2_ de final de corrente) e situações de circulação hiperdinâmica associados a espaço morto e/ou shunt possivelmente expliquem nossos resultados.

**CONCLUSÃO:**

Em pacientes com lesão pulmonar aguda, o débito cardíaco determinado pela reinalação parcial de gás carbônico difere dos valores medidos com termodiluição. Esta diferença se acentua com a maior gravidade da lesão pulmonar.

## INTRODUCTION

Thermodilution (TD_CO_) is a method for cardiac output measurement that was introduced by Ganz et al.,^1^ originally in patients with acute myocardium dysfunction. It is still the established clinical standard for measuring cardiac output. TD_CO_ is a widely available technique that is relatively simple to perform and allows for immediate results that can be reproduced at the bedside.^1-4^

In spite of its many positive aspects, thermodilution has some disadvantages. It requires deep vein access and the positioning of the distal extremity of the catheter in the pulmonary artery. These factors add morbidity to the method.^5-9^ Such problems provide good motivation for considering noninvasive methods for measuring cardiac output, such as the partial carbon dioxide rebreathing method (RB_CO_).

RB_CO_ is a noninvasive method and is therefore free from the risk of infection. It is simple, automatic, continuous and operator-independent. Its use is restricted to intubated and ventilated patients with a constant exhaled volume. Many authors have demonstrated a good correlation between cardiac outputs obtained by TD_CO_ and RB_CO_ in studies using animals,^10,11^ and also in patients submitted to anesthetic-surgical procedures without any evident lung injury.^12-18^

There is, however, evidence that suggests that, in the presence of lung injury, this method has some limitations.^19-22^ The aim of the present study was therefore to compare cardiac output measurements by thermodilution and partial carbon dioxide rebreathing in patients with acute lung injury at two levels of severity.

## METHODS

This was a comparative, prospective and controlled study, approved by the Ethics and Research Committees of the institutions involved. After informing and obtaining the consent of their legally responsible relatives, 20 patients were included: 15 men and 5 women, aged between 24 and 80 years (average of 46.42 years). These patients were submitted to mechanical ventilation to treat acute hypoxemic breathing insufficiency, characterized by PaO_2_/FiO_2_ of less than 300 mmHg. Invasive hemodynamic monitoring had previously been installed in these patients because of their hemodynamic instability. Patients who had chronic lung disease and spontaneous ventilation, and those undergoing techniques that did not assure constant ventilation, were excluded from the study.

During the study, volume-controlled ventilation was applied at peak and plateau pressures up to the limits of 45 cmH_2_O and 35 cmH_2_O, respectively. Comfort and adaptation to ventilation were assured by continuous sedation using midazolam and fentanyl. Patients were kept lying down, with the head elevated at 30 degrees. The infusion of solutions or vasoactive drugs was adjusted according to needs, on the basis of data supplied by conventional hemodynamic monitoring.

The lung injury score (LIS) was calculated from the fraction of inhaled oxygen measurements, positive final exhalation pressure, static complacence of the respiratory system and extent of pulmonary infiltrates on chest x-ray, as proposed by Murray et al. (1988).^23^ The patients were divided into two groups, LIS < 2.5 (group A, n = 11) and LIS > 2.5 (group B, n = 9). The main diagnoses and the lung injury scores (LIS) of the patients studied are shown in Table 1.

**Table 1:** Main diagnoses and lung injury scores (LIS) for the patients studied

Case	Main diagnoses	LIS
1	Bronchopneumonia + septic shock	2.75
2	Bronchopneumonia + congestive cardiac injury + septic shock	1.20
3	Acute abdominal perforation + peritonitis + septic shock	2.00
4	Acute myocardial infarction + cardiogenic shock	0.75
5	Acute myocardial infarction + cardiogenic shock	1.50
6	Acute abdominal perforation + peritonsillar abscess + septic shock.	2.00
7	Biliary peritonitis + septic shock	2.00
8	Abdominal wall abscess + bronchopneumonia + septic shock	3.00
9	Multiple trauma (acute thoracic trauma + long bone trauma) + hemorrhagic shock	3.50
10	Multiple trauma + hemorrhagic shock + multiple transfusion + acute renal failure	2.25
11	Digestive hemorrhage + hemorrhagic shock + multiple organ insufficiency	2.00
12	Bronchoaspiration + septic shock	2.75
13	Multiple trauma (diaphragmatic traumatic hernia + general abdominal-perineal injury) + hemorrhagic shock	2.75
14	Aspirative bronchopneumonia + acute cholecystitis + septic shock	3.00
15	Multiple trauma (cranial-encephalic trauma) + long bone fractures) + septic shock	1.25
16	Acute abdominal perforation + peritonitis + septic shock	2.25
17	Aspirative bronchopneumonia + septic shock	2.25
18	Multiple trauma + bronchopneumonia	2.70
19	Bronchopneumonia + acute/chronic renal insufficiency	3.00
20	Ulcer perforation + diffuse peritonitis + septic shock	2.75

For TD_CO_, a pulmonary artery catheter (Swan-Ganz Baxter Inc, USA) and an SDM-2010 monitor (Dixtal^®^, Manaus, Brazil) were used, with their position checked by chest x-ray and by observing the pressure curves that were generated. The cardiac output was obtained by injecting 10 ml of 0.9% saline solution at a temperature of between 0 and 5° C into the proximal orifice of the catheter, respecting a timing of two to four seconds. Four measurements were performed per hour, always at the end of expiration, with a maximum accepted difference between them of 10%. The average of the three most similar measurements was taken to be the definitive measurement. Among these 20 patients, 294 cardiac output determinations were made: 164 in group A and 130 in group B (ranging from 14 to 15 determinations per patient).

A NICO_2_^®^ monitor (Novametrix Medical Systems, Wallingford, Connecticut, USA) was used to determine RB_CO_. Before measuring, 30 minutes were spent on calibrating the sensor of the NICO_2_ capnograph, stabilizing the system and adjusting the rebreathing circuit, in accordance with the currently exhaled volume. The monitor signals when the measurements of exhaled gas are stable, by means of confidence intervals graded from 1 to 5. In this device, the measuring of cardiac output is completely automatic, with no interference from the operator.^24,25^

The curves of flow versus volume were used to assess and correct for the presence of leakages and/or airway secretion, factors that could have interfered with the measurement. Following this, arterial blood gas was collected, without rebreathing. The measurements of FiO_2_, PaO_2_, PaCO_2_ (ABL 500 Radiometer) and the concentration of hemoglobin were then recorded on the device. Four measurements were made: two right before and two right after TD_CO_, and the one that differed most from the others was not taken into consideration. The average of the three remaining measurements was accepted as the definitive measurement for analysis.

For the analysis of the results, the patients were divided into two groups, A and B, according to their LIS measurement, and the following tests were performed:

1. Fischer's exact test for 2 x 2 tables,^26^ with the aim of comparing groups A and B regarding their gender composition.
2. Mann-Whitney test for two independent samples,^26^ with the aim of comparing groups A and B regarding age.
3. Variance analysis for non-independent groups,^27^ separately for groups A and B, with the aim of checking the homogeneity of the measurements in triplicate for TD_CO_ and RB_CO_.
4. Linear regression,^27^ with the aim of studying possible relationships between the measurements using the TD_CO_ and RB_CO_ techniques. The regressions were calculated separately for groups A and B.
5. Bland-Altman test,^28,29^ with the aim of checking the agreement observed between the TD_CO_ and RB_CO_ techniques, performed independently for groups A and B.
6. Student's t test for paired data,^27^ with the aim of comparing the thermodilution (TD_CO_) and partial carbon dioxide rebreathing techniques, regarding the averages of the cardiac output measurements, performed separately for groups A and B.
7. Mann Whitney test for two independent samples,^26^ with the aim of comparing groups A and B, regarding the differences in percentages (Δ%) observed between the averages. The formula Δ% = RB_CO_ - TD_CO_/TD_CO_ x 100 was used to calculate Δ%. The level for rejection of the nullity hypothesis was set at 0.05 or 5%, and significant measurements were signaled with an asterisk.

## RESULTS

No significant differences were observed between groups A and B regarding age (averages: group A = 41.18 versus group B = 51.67 years; p = 0.87) or gender (group A: 9 men and 3 women; group B: 6 men and 3 women; p = 0.39).

Neither method, and neither group, showed any relevant difference between the measurements made in triplicate. The average and standard deviation of the cardiac output measurements that made up the triplicate for TD_CO_ (TD_CO1_, TD_CO2_ and TD_CO3_) and RB_CO_ (RB_CO1_, RB_CO2_ and RB_CO3_) were, respectively: group A – TD_CO_ = 8.58 ± 2.75 versus 8.61 ± 2.78 versus 8.60 ± 2.74 (p = 0.78); group A – RB_CO_ = 7.69 ± 2.78 versus 7.70 ± 2.78 versus 7.70 ± 2.74 (p = 0.93); group B – TD_CO_ = 7.50 ± 1.88 versus 7.52 ± 1.87 versus 7.53 ± 1.91 (p = 0.74); and group B – RB_CO_ = 5.77 ± 2.12 versus 5.76 ± 2.07 versus 5.77 ± 2.10, (p = 0.97).

**Figure 1:**
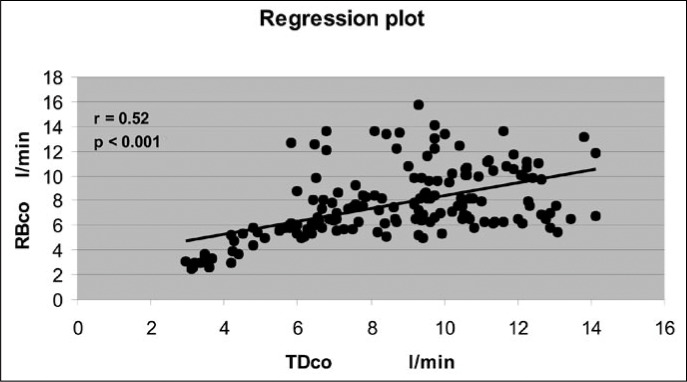
Linear regression and correlation of 164 cardiac output measurements via thermodilution (TDCO) and partial carbon dioxide rebreathing (RBCO), for eleven patients with acute respiratory insufficiency and lung injury score (LIS) of less than 2.5 (group A).

**Figure 2:**
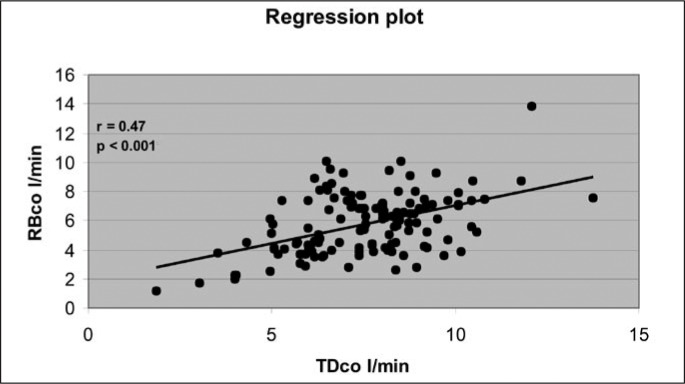
Linear regression and correlation of 130 cardiac output measurements via thermodilution (TD_CO_) and partial carbon dioxide rebreathing (RB_CO_), for nine patients with acute respiratory insufficiency and lung injury score (LIS) of more than 2.5 (group B).

Figures 1 and 2 show the regression and correlation between the measurements of TD_CO_ and RB_CO_ separately for groups A and B. In group A (n = 11), 164 paired measurements (range: TD_CO_ 3.17 to 13.80 l/min; RB_CO_ 2.50 to 13.63 l/min) were performed, with a correlation coefficient (r) of 0.52 (p = 0.001), and the calculated expression that relates the variables was: RB_CO_ = 3.2146 + 0.52150 TD_CO_. In group B (n = 9), with 130 measurements (range: TD_CO_ 1.87 to 13.77 l/min; RB_CO_ 1.17 to 13.80 l/min), the correlation coefficient (r) observed between the variables was 0.47 (p < 0.001), and the calculated expression was: RB_CO_ = 1.8544 + 0.52073 TD_CO_.

**Figure 3:**
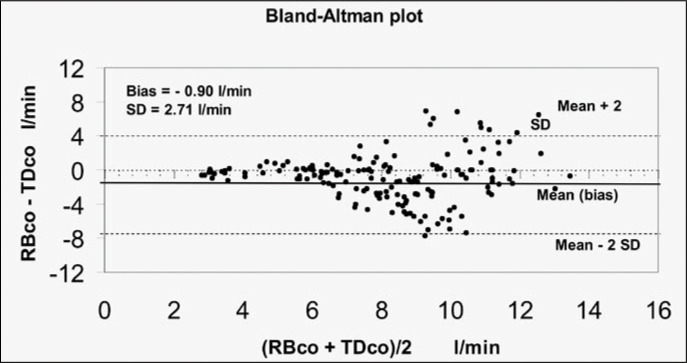
Agreement between 164 cardiac output measurements via thermodilution (TDCO) and partial carbon dioxide rebreathing (RB_CO_), for eleven patients with acute respiratory insufficiency and lung injury score (LIS) of less than 2.5 (group A). The solid line represents the average of the differences (bias), and the dotted lines define the agreement limits (95% confidence interval). SD = standard deviation.

**Figure 4:**
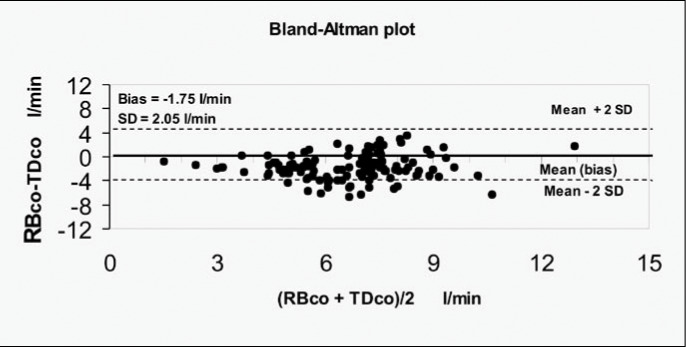
Agreement between 130 cardiac output measurements via thermodilution (TD_CO_) and partial carbon dioxide rebreathing (RB_CO_), for nine patients with acute respiratory insufficiency and lung injury score (LIS) of more than 2.5 (group B). The solid line represents the average of the differences (bias), and the dotted lines define the agreement limits (95% confidence interval). SD = standard deviation.

In Figures 3 and 4, the agreement between the two methods is displayed separately for groups A and B, via the Bland-Altman Test. In group A (n = 11), for a total of 164 paired measurements, the average of the differences (bias) between the two methods, compared with the average between RB_CO_ and TD_CO_, was - 0.90 ± 2.71 l/min (95% confidence interval, CI = - 1.14 to - 0.48). In group B (n = 9), for a total of 130 paired measurements, the average of the differences between the two methods, compared with the average between RB_CO_ and TD_CO_, was −1.75 ± 2.05 l/min (95% CI = −2.11 to −1.40).

The analysis of Student's t test for paired groups showed statistically significant differences between TD_CO_ and RB_CO_ for each group (group A: 8.60 ± 2.74 versus 7.70 ± 2.76; p = 0.000; and group B: 7.52 ± 1.87 versus 5.77 ± 2.07; p = 0.000) and between them (p = 0.000), as shown in Table 2.

**Table 2:** Calculations of the percentage differences D% [RBCO -TDCO/TDCO X 100] for the patients with acute respiratory failure and lung injury score (LIS) of less than 2.5 (group A) or more than 2.5 (group B), according to cardiac output measurements (average and standard deviation) via the thermodilution (TDCO) and partial carbon dioxide rebreathing (RBCO) techniques

		Group A			Group B	
	TD_CO_	RB_CO_	Δ%	TD_CO_	RB_CO_	Δ%
Average	8.60	7.70	-6.97	7.52	5.77	-22.01
Standard deviation	2.74	2.76	-	1.87	2.07	-

*Student's t test for paired groups (TD_CO_ versus RB_CO_): p_critical_ < 0.05. Group A (TD_CO_ > RB_CO_): p_calculated_ = 0.000. Group B (TD_CO_ > RB_CO_): p_calculated_ = 0.000; Mann-Whitney test (Group A versus Group B) for Δ%: p_calculated_ = 0.00; p_critical_ = 0.05*

## DISCUSSION

Application of the Fick principle in indirect methods using total or partial carbon dioxide rebreathing has led to new alternatives for measuring cardiac output.^30-32^ The technique of partial carbon dioxide rebreathing, using a NICO_2_^®^ monitor, is the variant of the Fick method for noninvasive measurement of the effective pulmonary capillary blood flow (PCBF) that the cardiac output infers. This technique was first described by Gedeon et al.^33^ and later expanded by Capek and Roy.^34^ With the partial rebreathing technique, variations in VCO_2_ and ETCO_2_ (end-tidal CO_2_) occur in response to changes in ventilation and allow Fick's differential equation to be applied.

Fick's differential equation for CO_2_ shows that:


$$\text{PCBF}=\frac{{\text{VCO}}_{2}}{{\text{C}}_{\text{v}}{\text{CO}}_{2}-{\text{C}}_{\text{a}}{\text{CO}}_{2}}$$

Where: PCBF = pulmonary capillary blood flow; VCO_2_ = elimination of CO_2_; C_v_CO_2_ = mixed venous CO_2_ content; C_a_CO_2_ = arterial CO_2_ content.

When applied with or without rebreathing, this gives:


$$\text{PCBF}=\frac{{\text{VCO}}_{2\text{N}}}{\left({\text{C}}_{\text{v}}{\text{CO}}_{2\text{N}}-{\text{C}}_{\text{a}}{\text{CO}}_{2\text{N}}\right)}=\frac{{\text{VCO}}_{2\text{R}}}{\left({\text{C}}_{\text{V}}{\text{CO}}_{2\text{R}}-{\text{C}}_{\text{a}}{\text{CO}}_{2\text{R}}\right)}$$

Where: PCBF = pulmonary capillary blood flow; VCO_2N_ = CO_2_ elimination without rebreathing; VCO_2R_ = CO_2_ elimination with rebreathing; C_v_CO_2N_ = mixed venous CO_2_ content without rebreathing; C_v_CO_2R_ = mixed venous CO_2_ content with rebreathing; C_a_CO_2N_ = arterial CO_2_ content without rebreathing; C_a_CO_2R_ = arterial CO_2_ content with rebreathing.

Combining these to form Fick's differential equation, this gives:


$$\begin{array}{c} \text{PCBF}=\frac{{\text{VCO}}_{2\text{N}}-{\text{VCO}}_{2\text{R}}}{\left({\text{C}}_{\text{V}}{\text{CO}}_{2\text{N}}-{\text{C}}_{\text{V}}{\text{CO}}_{2\text{R}}\right)-\left({\text{C}}_{\text{a}}{\text{CO}}_{2\text{N}}-{\text{C}}_{\text{a}}{\text{CO}}_{2\text{R}}\right)}\\ =\frac{\Delta {\text{VCO}}_{2}}{\Delta {\text{C}}_{\text{a}}{\text{CO}}_{2}}=\frac{\Delta {\text{VCO}}_{2}}{\text{S}\Delta {\text{ETCO}}_{2}} \end{array}$$

Where: ΔVCO_2_ = difference in elimination of CO_2_ in phases _R_ and _N_; ΔC_a_CO_2_ = difference between arterial CO_2_ content in phases _R_ and _N_; S = slope of the CO_2_ dissociation curve; Δ ETCO_2_ = difference between CO_2_ exhaled at the end of exhalation in phases _R_ and _N_.

Considering that the concentrations of CO_2_ in mixed venous blood do not significantly change during the 50 seconds of the rebreathing period, they are canceled out in the mathematical formula and are therefore unnecessary for the calculation of the PCBF.^33^ This allows PCBF to be obtained by means of noninvasive parameters. The ΔETCO_2_ reflects the ΔPaCO_2_.

The NICO_2_^®^ monitor includes a device consisting of a valve adapted to the rebreathing circuit and a combined sensor for CO_2_ and flow. This is located between the tracheal tube and the Y of the rebreathing circuit. Every three minutes, with the valve activated, a rebreathing volume is added to the circuit, causing the patient to inhale a fraction of the exhaled gases.

The VCO_2_ is calculated via the mathematical integration of flow and signs of CO_2_, which are measured practically at the same point in the patient's airway, thereby ensuring maximum precision. The process takes place in three phases: firstly, for 60 seconds, the rebreathing valve is positioned so that the exhaled gases do not go through the additional circuit and the measurements of VCO_2_, ETCO_2_ and PaCO_2_ represent the baseline measurements. Next, the valve is opened and the currently exhaled volume is deviated to the circuit, for 50 seconds, causing rebreathing of the gases, increase in PaCO_2_ and ETCO_2_ and reduction in VCO_2_. Thirdly, the rebreathing valve returns to its initial position and the gases are eliminated directly into the circuit, thus returning to the baseline measurements.^25^

Variations in VCO_2_ and ETCO_2_ reflect only the gas exchanges, which happen in the perfused and ventilated areas of the lung. This makes it necessary to include a correction factor for the blood flow shunted away from the lungs (shunt). The computer estimates the shunt fraction based on data (FiO_2_ and PaO_2_), arterial saturation of O_2_ (pulse oximetry) and Nunn's iso-shunt graphs. The cardiac output is the result of adding together the PCBF and the estimated shunt.

Several investigators have tried, using different models and clinical conditions, to analyze the correlation and agreement between the cardiac output measurements obtained via RB_CO_ and TD_CO_. From studies performed on humans without lung injury^12-17^ and laboratory animals with normal lungs,^10^ it is possible to conclude that RB_CO_ reflects TD_CO_. In experimental models of induced lung injury in dogs, Johnson et al.^11^ noticed coincidence between these two methods. These results were not confirmed by Maxwell et al.^22^ in induced thoracic trauma in pigs, or by Gama de Abreu et al.^35^ in sheep. Lack of agreement between RD_CO_ and TD_CO_ after extracorporeal circulation has also been observed in humans.^21,36^

To analyze these divergences we have to consider some aspects of the methodology involved that were not always explicit in the research mentioned above. The present study has attempted to check the reproducibility of the data obtained via the two methods, as well as treating them statistically in order to define their behavior at two clinical conditions differing in severity. Finally, the reasons that might explain the differences found are discussed.

The lack of agreement can be attributed to the lung injury, but before assessing differences, correlation, regression and agreement, it was necessary to assess the dispersion of the measurements that made up the triplicates for TD_CO_ and RB_CO_, for groups A and B separately. Analysis of the results from this confirmed the low variability among data, which allowed the use of the average as a definitive measurement for comparing TD_CO_ and RB_CO_.

There was a poor positive correlation between the methods studied, among the patients in group A (r = 0.52; p < 0.001) and in group B (r = 0.47, p < 0.001). Nevertheless, correlations and linear regression analyses (calibration statistics), when used singly, may be misleading. That was the reason for using the analysis proposed by Bland and Altman in 1986, in which a standard for assessing the agreement between two measurement methods for the same parameter is utilized.^29,37^

In group A (Figure 3), the average of the differences between the two methods, in relation to the average of the measurements obtained with both methods, showed considerable discordance (-0.9 ± 2.71 l/min; 95% CI = −1.14 to - 0.48) between RB_CO_ and TD_CO_. This difference is enhanced when these methods are applied to patients with lung injury that is even more severe (group B). In group B, the average of the differences was −1.75 ± 2.05 l/min (95% CI = −2.11 to −1.4) (Figures 3 and 4).

The analysis of the results performed using Student's t test and the Mann-Whitney test, applied to the 11 patients in group A (164 paired measures) and 9 patients in group B (130 paired measures), confirmed the existence of significant differences between the techniques (Table 2).

The discordance found here surpassed the acceptable limits, according to standards currently used in the medical literature. The criteria used in the literature for checking errors and statistical agreement between measurements of cardiac output were recently reassessed in a meta-analysis.^38^ Agreement limits of less than 1 l/min, percentage limits of less than 20% and findings in which more than 75% of measurements varied less than 20% from the average were considered to be clinically acceptable. From the above, it is evident that the presence of lung injury hinders the performance of RB_CO_.

Although the analysis of the reasons that produced such lack of agreement between RB_CO_ and TD_CO_ was not the main objective of the present study, some comments on this matter are necessary. RB_CO_ presumes that, during cardiac output measurements, the CO_2_ content in mixed venous blood remains steady and can therefore be eliminated from the mathematical formula, a fact that was confirmed in the study by Nilsson et al.^21^ In spite of finding small increases in CO_2_ in mixed venous blood during the final phase of the rebreathing period, these authors concluded that the changes noted earlier did not render the method unfeasible, on account of their reduced magnitude.

In the presence of lung injury, the lung shunt, which is underestimated by RB_CO_, could be one of the reasons for the discordance observed between this method and TD_CO_. Nilsson et al.^21^ found discrepancies between the shunt calculated using samples of arterial gases (0.20 ± 0.05) and the shunt estimated via NICO_2_^®^ from SpO_2_, PaO_2_ and Nunn's iso-shunt curves (0.14 ± 0.11). Factors such as the imprecision of pulse oximetry and the behavior of the relationships between SpO_2_ and PaO_2_ could influence the results thus calculated.^39^ This was, however, contested by Haryadi et al.,^10^ who estimated that the influence of intrapulmonary shunt error on the cardiac output measurements via the rebreathing technique was not relevant.

Differences between TD_CO_ and RB_CO_ could further be attributed to errors in estimating CaCO_2_ from ETCO_2_.^21,35,40^ In a recent study, Gama de Abreu et al.^35^ concluded that RB_CO_, when checked by a noninvasive algorithm, underestimated cardiac output systematically. This happened mainly in hyperdynamic situations associated with dead space and/or increased shunt, which might possibly explain our results. The reasons suggested for this were the presence of intermittent recirculation of CO_2_ and systematic errors associated with both methods of CO_2_ measurement: blood gas analysis for PaCO_2_ and absorption of infrared light for ETCO_2_. The authors demonstrated the possibility of improving the RB_CO_ technique using a new algorithm that considers the arterial blood gas measurements in order to correct possible variations in PaCO_2_ and estimated PCBF. However, in consonance with the results of our study, Gama de Abreu et al. had the feeling that RB_CO_ did not perform satisfactorily in situations of hyperdynamic circulation and increased dead space.^35^

## CONCLUSIONS

Under the conditions of this study, the results obtained allow us to conclude that, in patients with acute lung injury, the cardiac output determined by partial rebreathing of CO_2_ differs from those measurements obtained by thermodilution. This divergence is enhanced, the more critical the lung injury is.
